# Authors’ response to correspondence for EPI-STREP-064 publication

**DOI:** 10.1007/s00431-018-3180-4

**Published:** 2018-06-14

**Authors:** Amparo Escribano Montaner, Juan García de Lomas, José Ramón Villa Asensi, Oscar Asensio de la Cruz, Olga de la Serna Blázquez, Mikel Santiago Burruchaga, Pedro Mondéjar López, Alba Torrent Vernetta, Yang Feng, Melissa K. Van Dyke, Janet Reyes, Pilar Garcia-Corbeira, Carla A. Talarico, on behalf of the EPI-Strep-064 study group, Silvia Castillo Corullón, Maribel Barrio Gómez de Agüero, Manuel Sánchez Solís, Antonio Moreno Galdó

**Affiliations:** 1 Pediatric Pneumology and Cystic Fibrosis Unit, University Clinic Hospital of Valencia, University of Valencia, Av. de Blasco Ibáñez, 13, 46010 Valencia, Spain; 2 Department of Microbiology, School of Medicine and University Hospital, University of Valencia, Av. de Blasco Ibañez 17, 46010 Valencia, Spain; 3 Pediatric Department, Niño Jesús University Hospital for Children, Calle Menéndez Pelayo, 65, 28009 Madrid, Spain; 4 Pediatric Pulmonology Unit, University Hospital Parc Tauli de Sabadell, Parc Taulí, 1, 08208 Sabadell, Barcelona Spain; 5 Pediatric Department, Hospital La Paz, Paseo de la Castellana, 261, 28046 Madrid, Spain; 6 Pediatric Pneumology and Cystic Fibrosis Unit, Cruces University Hospital, Plaza de Cruces, S/N, 48903 Barakaldo, Vizcaya Spain; 7 Pediatric Pulmonology and Cystic Fibrosis UnitVirgen of Arrixaca University Hospital, Murcia, Spain; 8 Paediatric Pulmonology and Cystic Fibrosis UnitVall d’Hebron University Hospital, Barcelona, Spain; 9 Ningyang Group Co., Limited, C/O GSK, Wavre, Belgium; 10 GSK, Collegeville, Collegeville, PA USA; 11 GSK, Parque Tecnológico de Madrid, Tres Cantos, Spain; 12 GSK, Rockville, Maryland, USA

Dear Drs. Hare, Grimwood, and Chang,

We sincerely thank you for your correspondence and appreciate you pointing out the unintended error in our publication’s citation. We agree with your response and feedback.

To clarify the point that there is heterogeneity in the literature about the use of NPS to understand lower lung microbiota in disease, we have added the following references to our sentence:

“Several studies assessed a possible correlation between BALF and nasopharyngeal swab (NPS) specimens in terms of chronic LRTI etiology, with inconsistent results [1–5].”

Carla A. Talarico, on behalf of the co-authors
